# Biological Activity of Thyme White Essential Oil Stabilized by Cellulose Nanocrystals

**DOI:** 10.3390/biom9120799

**Published:** 2019-11-28

**Authors:** Jonghyun Shin, Kyunga Na, Sungchul Shin, Seon-Mi Seo, Hye Jung Youn, Il-Kwon Park, Jinho Hyun

**Affiliations:** 1 Department of Biosystems and Biomaterials Science and Engineering, Seoul National University, Seoul 08826, Korea; sjh811@snu.ac.kr (J.S.); sungssc@snu.ac.kr (S.S.); 2 Research Institute of Agriculture and Life Sciences, Seoul National University, Seoul 08826, Korea; ellen@snu.ac.kr (K.N.); popcon24@naver.com (S.-M.S.); page94@snu.ac.kr (H.J.Y.); parkik1@snu.ac.kr (I.-K.P.); 3 Department of Forest Sciences, Seoul National University, Seoul 08826, Korea

**Keywords:** thyme white essential oil, biological activity, Pickering emulsion, cellulose nanocrystal

## Abstract

Cellulose nanocrystals (CNCs) are produced by sulfonic acid hydrolysis and used for the formation of Pickering emulsion (PE) with thyme white essential oil (EO). Highly volatile and hydrophobic thyme white is encapsulated in PE by the amphiphilicity of CNCs. Encapsulation of EO in a CNC shell is determined by confocal microscopy with distinct fluorescent labelling. The amount of CNC affects the size distribution of PE, and the emulsion stability is confirmed by rheological property. The antimicrobial activity of the emulsion is evaluated against *Escherichia coli* and *Staphylococcus aureus* by minimal inhibitory concentration and minimum bactericidal concentration. The larvicidal activity is also investigated against *Aedes albopictus* by dispersing the emulsion in water.

## 1. Introduction

Plant essential oils (EOs) are natural substances that have been actively studied in the fields of pharmaceuticals, packaging and cosmetics for reducing microbial contamination [1,2,3,4]. EOs contain several bioactive ingredients, including alcohols, phenols, terpenes, esters, and others [5,6], and show strong antimicrobial activities. Thyme essential oil is a well-known substance showing antimicrobial activity against gram (−) and gram (+) bacteria compared with other plant EOs [7,8]. It also presents larvicidal activity against insects and could be a potential matter for mosquito-controlling agent.

However, EO has a low surface energy and a high volatility, which makes it difficult to use EOs in an aqueous phase [4]. The low surface energy of EO causes phase separation with aqueous solutions, and the high volatility of EO reduces the sustainability and stability of biological activity. For this reason, volatile EOs need to be encapsulated in the form of an emulsion that is dispersible in water.

The Asian tiger mosquito, *Aedes albopictus* Skuse, is a vector insect of dengue virus (DENV) and chikungunya virus (CHIKV), and has spread quickly to new locations from its native geographical origin [9]. *Ae. albopictus* management has mainly been dependent on various synthetic pesticides around the world [10,11]. However, the continued use of synthetic pesticides has caused several side effects, such as environmental and human health concerns and undesirable effects on natural enemies, non-targeted organisms [12,13]. Another problem is that the resistance of *Ae. albopictus* to synthetic pesticides has been reported in several countries [10,11]. Recently, plant EOs and their constituents are considered to be good sources for mosquito-controlling agents. Larvicidal activities of many plant EOs against *Ae. albopictus* have been reported [14,15,16,17,18]. Development of proper formulation is another research field for practical use of plant EO-based mosquito larvicides. Nanoemulsion-based formulation have been reported to improve efficacy, stability and solubility of plant EOs in water [19].

Pickering emulsions (PEs) are one method for encapsulating a phase separating liquid phase with surface active particles [20,21,22]. PE stabilizes biphases with different surface energies using amphiphilic particles instead of a low molecular weight linear surfactant. Cellulose nanocrystals (CNCs) are appropriate particles for PEs because they have a high aspect ratio of crystalline fibrils, as well as amphiphilicity [23,24].

The stability and morphology of PEs are closely related with the properties of emulsifying particles. A high aspect ratio CNC provides high structural stability to the emulsion and colloidal dispersion in the aqueous solution. Recently, emulsions stabilized with cellulose-based particles were demonstrated using microfibrillar cellulose, nanofibrillar cellulose and CNCs [25,26,27,28,29,30]. Such a cellulose-based stabilizer for PEs is advantageous over synthetic or inorganic nanoparticles due to biocompatibility, degradability and cost issues [31].

In particular, CNCs are attractive as stabilizers compared with other types of cellulose particles such as sphere and nanofibers due to the better control in morphology and reproducibility of emulsion formation [27,32,33]. The rod-like CNCs tend to pack orderly because of the strong capillary forces, and space steric hindrance is structured at the interface inducing the resistance to coalescence of emulsions. On the contrary, a low aspect ratio of CNCs arrange in a disorderly fashion on the emulsion droplet and desorb easily from the interface.

Here, a volatile EO is encapsulated in a PE using nature-derived CNC particles, and the EO solubility can be improved by dispersion in water. The amount of CNC and emulsifying agent in the process affects the size distribution of PE particles as well as the emulsion stability. Since sustained biological activity is one of the critical issues in ensuring reliable quality and reduced cost in industry, a long-term evaluation of biocidal effects using CNC stabilized PE of EO (CNC/EO) is required.

## 2. Materials and Methods

### 2.1. Materials

Cotton CNCs were prepared with filter paper (Whatman, grade 2, Kent, UK) by the hydrolysis of sulfuric acid (Junsei Chemical Co, Ltd., 95.0%(*w*/*w*) purity, Tokyo, Japan) [34,35]. Thyme white EO (Korea Similac, Pochun, Korea) was purchased and used without purification. Temephos (95.6%) was purchased from Sigma-Aldrich (Milwaukee, WI, USA). The bacterial incubation medium was prepared using Mueller Hinton (MH) broth (BD Difco, Bergen, NJ, USA.) and Biochemie micro agar (Duchefa Biochemie, Amsterdam, The Netherlands).

### 2.2. Preparation and Characterization of Sulfated CNC

Cotton filter paper was ground at 1800 rpm for 5 min in a blender to increase the surface area available to the sulfuric acid. Acid hydrolysis was then carried out at 45 °C for 1 h in 60% (*w*/*w*) sulfuric acid. The treated solution was centrifuged repetitively at 6000 rpm for 10 min until the CNC sediment was not observed. Then, the collected solution was enclosed in a MWCO 15 kDa cellulose dialysis membrane (Spectra/Por, Breda, The Netherlands) and dialyzed in running deionized water for one week.

The CNC suspension was diluted to 0.1% (*w*/*v*) for calculating length, width, and thickness with a transmission electron microscope (TEM, JEM1010, JEOL, Tokyo, Japan). The CNC was loaded on a glow-discharged carbon copper grid and was then treated with a 10 µL uranyl acetate solution (1% *w*/*v*) for negative staining. The grids were dried at room temperature for 30 min, and the dried grid was used to obtain images of CNCs [36]. The length and width of the CNCs were measured from the TEM images using the ImageJ program, and the CNC sample number was 300.

The sulfate content of CNC samples was determined by conductometric titrations. 35 mg of CNC samples were suspended in 15 mL of a 0.1 M hydrochloric acid solution and stirred for 10 min. After 10 min of stirring, the solution was titrated with 0.1 M NaOH [29,37,38]. The conductivity of the solution was measured by a conductometer (SevenGo Duo pro, Mettler Toledo Inc., Columbus, OH, USA) during titration.

### 2.3. Preparation of CNC-Stabilized PE

CNC/EO PE was prepared by adding 45, 90, 135 and 180 mg per 1 mL of EO to observe the state of the PE as a function of various CNC contents. EO was fixed at 10 wt% of the total solution, then the CNC was added, and tip-sonication (VCX 130, Sonics & Materials, Inc., Newtown, CT, USA) was performed at 50% amplitude for 30 s.

To evaluate the colloidal stability of PEs, a glass vial containing 10 wt% thyme white PEs was placed in a Turbiscan (Turbiscan Lab Expert, Formulaction, Toulouse, France), and the dispersion stability of the emulsion was observed for 24 h at room temperature. The Turbiscan Stability Index (TSI) was calculated using the Turbiscan Easy Soft program installed in the equipment.

PE suspension solutions were visualized with a polarized light microscope (LV100, Nicon, Tokyo, Japan) in a dark-field mode. The prepared PE was diluted 10 times with distilled water and stirred for about 1 min. Then, 100 µL of diluted PE suspension solution was pipetted and dropped onto a slide glass and covered with a cover glass to prevent the vaporization of EO. The diameter of PE particles was measured using the ImageJ (1.52a, National Institutes of Health, Bethesda, MA, USA) program.

CNC and thyme white EO were stained with Calcofluor white (Sigma Aldrich, St. Louis, MI, USA) and Nile red (Sigma Aldrich, St. Louis, MI, USA), respectively. The stained CNC and thyme white EO were tip-sonicated for 1 min and stored at room temperature for 3 d prior to observations. The fluorescently labeled PE was diluted 10 times with distilled water and stirred for about 1 min. Then, 100 µL of a labeled PE solution was pipetted and dropped onto the slide glass and covered with a cover glass to prevent the vaporization of EO. Observation of the labeled PE was performed with a 40 × oil immersion objective lens using a confocal laser scanning microscope (LSM710, Carl Zeiss, Overkochen, Germany).

Rheological measurements were carried out using a digital rheometer (MARS III, Thermo Scientific, Newington, NH, USA) equipped with a 35 mm plate-plate geometry and a temperature controller [39]. Specifically, 100 µL of the PE was dropped on the plate for dynamic viscoelastic measurements. The gap size of the plate-plate was then adjusted to 0.1 mm, and mineral oil was dropped around the plate to prevent evaporation of the emulsion. The frequency sweep measurement was performed at 0.5% strain in the range of 0.1 to 10 Hz [27,28].

### 2.4. Gas Chromatography (GC-FID) and Gas Chromatography–Mass Spectrometry (GC-MS)

The chemical analysis of thyme white essential oil was conducted using an Agilent 7890B (Agilent Technologies, Santa Clara, CA, USA) equipped with a flame ionization detector (FID). We used a DB-5MS (30 m × 0.25 mm i.d., 0.25 µm film thickness, Agilent, CA, USA) and HP-innowax column (30 m × 0.25 mm i.d., 0.25 µm film thickness, Agilent, CA, USA). The oven temperature was programmed as isothermal at 40 °C for 6 min, raised to 250 °C at the rate of 6 °C/min. The flow rate of the carrier gas (nitrogen) was 1.0 mL/min. The retention indices were calculated in relation to a homologous series of n-alkanes (C10-C29) under the same GC operating conditions. The constituents of thyme white essential oil were further analyzed using a gas chromatograph (Agilent 7890B)-mass spectrometer (Agilent 5977B MSD, Agilent Technologies, Santa Clara, CA, USA) (GC-MS) with an DB-5 MS column (30 m × 0.25 mm i.d., 0.25 µm film thickness, Agilent, CA, USA). The oven temperature program was the same as that used for the GC-FID analysis with helium as the carrier gas at a flow rate of 1.0 mL/min. Ionization was achieved using electron impact (70eV, source temperature 230 °C), and the scan range was 25–800 amu. Most compounds of thyme white essential oil were identified by comparing the mass spectra with those of authentic samples in the NIST MS library.

### 2.5. Evaluation of Antibacterial Performance

All antimicrobial tests were carried out using MH broth and Biochemie micro agar following the protocol of Wiegand et al. [40] Briefly, a liquid medium was prepared by addition of 15 g/L micro agar to a 21 g/L MH broth solution. After sterilization of a medium solution at 121 °C for 20 min, the medium solution was added to a 15 mL petri dish (15 mm × 100 mm) and solidified at room temperature. Prior to the experiment, the solid medium was incubated at 37 °C for 30 min to identify the contamination. The antimicrobial activity of EO was evaluated using *Escherichia coli* (*E. coli*) and *Staphylococcus aureus* (*S. aureus*).

The CNC/EO PE and thyme white only were added at 0.25, 0.5, 1, 2 and 4 µL/mL to the liquid medium containing 1 OD_600_/mL of microbial solution, and it was incubated at 37 °C while shaking. To measure the minimal inhibition concentration (MIC) and minimum bactericidal concentration (MBC) of CNC/EO PE and thyme white only solution [41,42], 1 mL of each solution was sampled. The absorbance of sampled solutions was measured at 600 nm with a microplate reader (Synergy HT, Bio Tek Instruments, Winooski, VT, USA). Then, the sampled solution was diluted 1000 times, spread on a solid medium, and incubated at 37 °C for 3 days, subsequently. The same concentration of Penicillin-Streptomycin solution (Life technologies, Carlsbad, CA, USA) was added to the liquid medium as a positive control sample.

### 2.6. Larvicidal Activity Test

We maintained *Aedes albopictus* cultures in the laboratory without exposure to any insecticides at 26 ± 1 °C, with a relative humidity of 60 ± 5%, under a 16:8 h light:dark cycle. A live mouse in a steel cage was supplied for blood under a Korea National Institute of Health Institutional Animal Care and Use Committee (KCDC-020-11-2A) protocol approved for this study. Larvae were reared in plastic pans (24 × 35 × 5 cm) containing sterilized food and water. Thyme essential oil dissolved in ethanol and CNC-based formulation dissolved in water at treated concentration was prepared, and one milliliter of thyme oil was applied in 200 mL of water in 270 mL paper cups. Ten early third instar *Ae. albopictus* larvae were used in each treatment. A separate set of cups that received 1 mL of ethanol or CNC only served as the controls. Treated and control larvae were kept at 26 ± 1 °C, with a relative humidity of 60 ± 5%, under a 16:8 h light:dark cycle, and larval mortality was investigated 48 h after treatment. All treatments were replicated 4 times.

## 3. Results and Discussion

The chemical compositions of thyme white essential oil are shown in Table 1. The most abundant compound in thyme white essential oil was *p*-cymene (38.14%) followed by thymol (35.82%), limonene (7.93%), *α*-pinene (7.22%), and linalool (3.57%). Other compounds were less than 2% of the oil. Thymol and *p*-cymene were also identified as two main components of thyme white essential oil in prior study [43]. Although there were a few differences in chemical composition of thyme white essential oil between our and prior studies, the major components were the same. Chemical composition of plant essential oil could vary according to dates of harvest, storage period, extraction method, or climate [44].

It is known that the negatively charged CNC forms more stable and uniform emulsions due to the restricted aggregation between CNCs [45]. Here, the CNC had a negative charge on the surface resulting from the hydrolysis of pulp with the sulfuric acid [28,29,46]. The degree of sulfate substitution was 0.40 as obtained by the conductometric titration. The value was similar to those of other literatures confirming that the anionic sulfate half ester group (OSO_3_^−^) was substituted to CNC [28,29,46].

**Figure 1:**
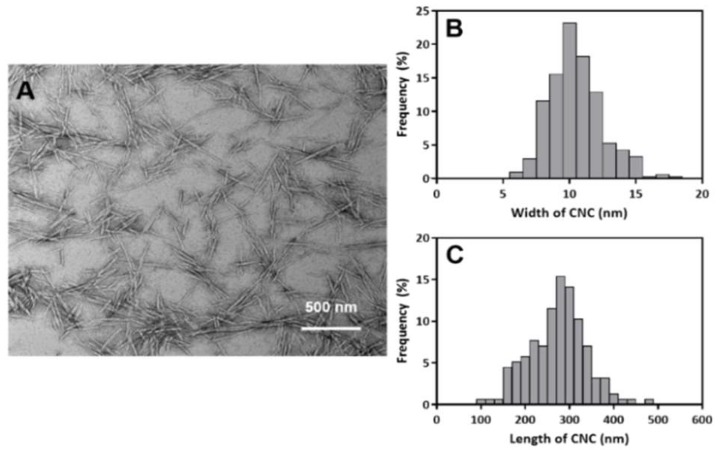
Morphological characteristics of CNCs. (**A**) TEM image of CNCs. (**B**) Width and (**C**) length distribution of CNCs. N = 300.

**Figure 2:**
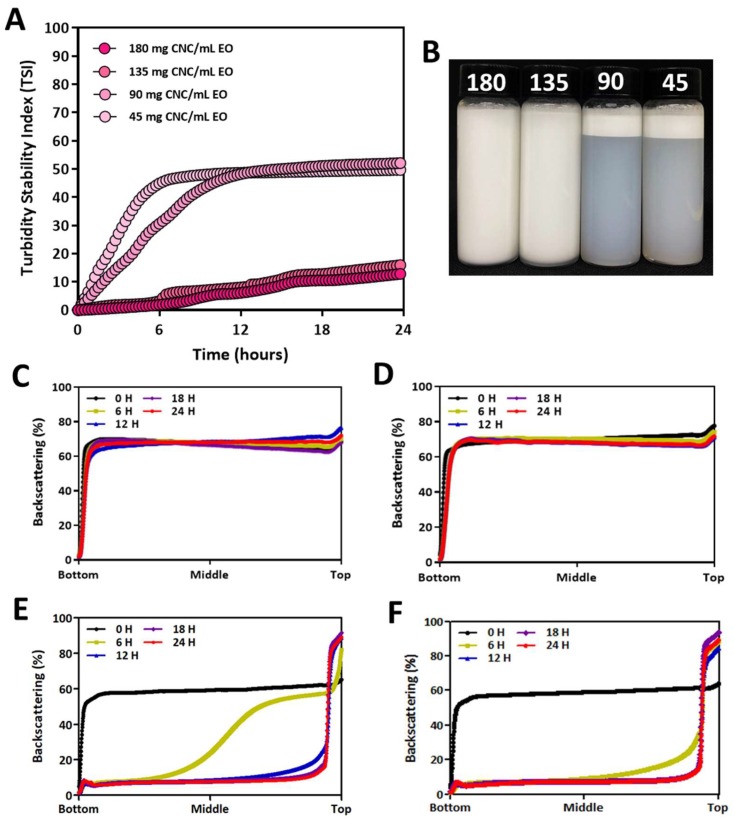
Colloidal stability of PE of CNC/EO prepared at different contents of CNCs. (**A**) TSI change for PEs for 24 h. (**B**) Photograph of the vials containing PEs after storage for 24 h. The numbers mean the CNC contents for 1 mL of EO, unit: CNC mg/mL EO. (**C**–**F**) Backscattering at the different vertical regions of the vial containing PE prepared with different contents of CNCs. (**C**) 180 mg CNC/mL EO, (**D**) 135 mg CNC/mL EO, (**E**) 90 mg CNC/mL EO, (**F**) 45 mg CNC/mL EO.

The produced sulfated CNC showed a typical needle-like morphology of 10 ± 2 nm in width and 274 ± 64 nm in length (Figure 1). A PE of CNC/EO was prepared by sonicating the mixture solution of thyme white and CNC suspension in water. The color of the solution changed to white, and the stability of the PE was determined by TSI values for 24 h (Figure 2A). Typically, TSI values varied from 0 to 100, and emulsions with a lower TSI value are more stable. The TSI values decreased as the content of CNC increased. The TSI values of 135 and 180 mg CNC/mL EO PEs were less than 12 while the TSI values of 45 and 90 mg CNC/mL EO PEs were above 50. The visual appearance of the emulsions showed significant differences depending on the CNC contents after storage for 24 h (Figure 2B). Usually, the light scattering of stabilized emulsions is high, and the transmission rate is very low through all the regions in the solution. However, transmission of light increased with the lower content of CNC emulsions because the emulsion particles were destroyed, and subsequent phase separation between EO and water occurred. This resulted from the insufficient content of emulsifying CNCs. The less covered EO was exposed to oil parts and the exposed parts were coalesced. The exposed oil flocculation moved to the aqueous surface and formed a floating creamy layer at the upper region [25,29,47].

The backscattering of light through each emulsion is shown in Figure 2C–F. The stability of emulsions was sustained without any significant phase separation with 135 and 180 mg CNC/mL EO emulsions during storage for 24 h (Figure 2C,D). In contrast, phase separation was observed in 6 h for emulsions prepared with 45 and 90 mg CNC/mL EO compositions (Figure 2E,F). This demonstrated that there is a critical concentration of CNCs required for completely covering EO and ensuring the long-term stabilization of CNC-based PEs. These results suggest that 135 mg CNC/mL EO could be close to the critical concentration of CNC/EO emulsions.

**Figure 3:**
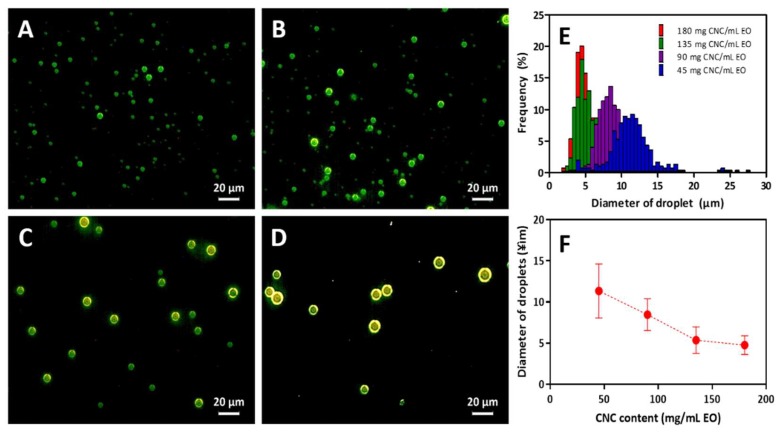
Size distribution of CNC/EO PE prepared at the different contents of CNCs. Dark-field microscopic images of PE prepared at (**A**) 180 mg CNC/mL EO, (**B**) 135 mg CNC/mL EO, (**C**) 90 mg CNC/mL EO and (**D**) 45 mg CNC/mL EO. (**E**) Distribution of the sizes of emulsions at different contents of CNCs. (**F**) Average diameter of droplets as a function of the content of CNCs. N = 300, error bar = SD.

Spherically shaped emulsion particles were observed with a dark field microscope as shown in Figure 3A–D. The dark-field microscopic image clearly shows the contours of solid particles, as well as their size distribution. The shell structure implies that the liquid phase was enclosed in the solid phase. The shell consisted of CNC particles because light scattering occurred at the edge of the solid particles. Thicknesses of the shell were similar regardless of the CNC/EO compositions, meaning that the assembly of CNCs was regular and uniform. The size of the PE decreased as the CNC content increased. The average shell size of the 45 mg CNC/mL EO PE was about 14 µm, and a broad size distribution was observed. It decreased to about 5 μm for 135 and 180 mg CNC/mL EO PEs with comparatively narrower size distributions (Figure 3E,F).

**Figure 4:**
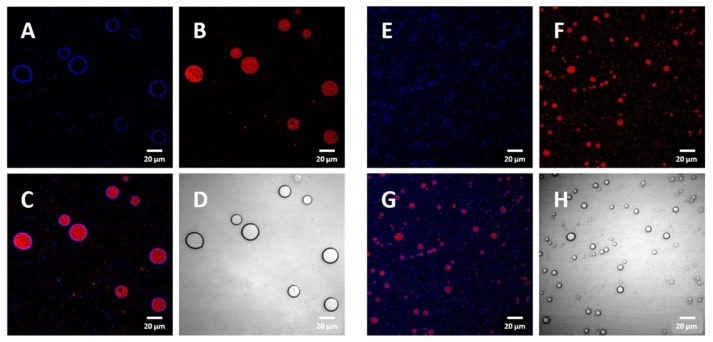
Confocal microscopic images of CNC/EO PEs. (**A**) CNCs labeled with blue fluorescent dye, (**B**) EO labeled with red fluorescence dye, (**C**) combined confocal image of A and B, and (**D**) bright field optical image with 90 mg CNC/mL EO. (**E**) CNCs labeled with blue fluorescent dye, (**F**) EO labeled with red fluorescent dye, (**G**) combined confocal image of (**E**) and (**F**), and (**H**) bright field optical image with 135 mg CNC/mL EO.

The enclosure of liquid EO in the CNC shell was confirmed with confocal microscope images (Figure 4). CNCs were labeled with blue fluorescent dye and were observed only at the boundary of the emulsion particles (Figure 4A,C). Meanwhile, the EO labelled with red fluorescent dye was observed at the inner region of the emulsion (Figure 4B,C). Smaller-sized EO droplets were contained in the CNC PE with a higher content of CNCs in the emulsion (Figure 4E–G). The emulsion particles were spherical and were well dispersed in the aqueous phase without aggregation (Figure 4D,H).

**Figure 5:**
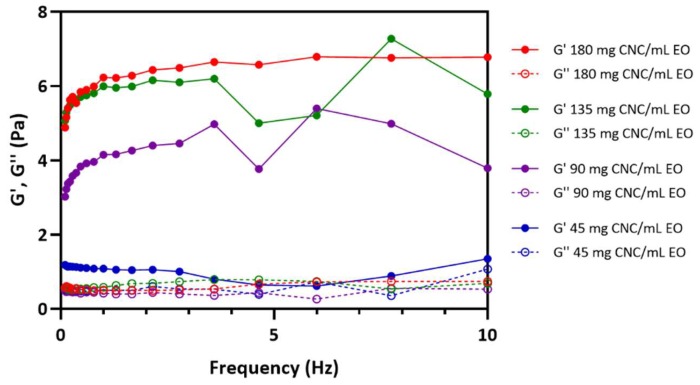
Rheological properties of CNC/EO PEs

The rheological properties of the CNC/EO emulsion were investigated as a function of the CNC content (Figure 5). The dynamic mechanical properties of the emulsions showed that the elastic storage modulus (G′) was higher than the viscous storage modulus (G″) in the frequency range of 0.1 to 10 Hz, and the PEs appeared to be a gel in this range.

PEs are very sensitive to shear forces. As the shear occurs, the network structure of the PE collapses, and the emulsions combine to increase the size. As the size of the PE increases due to the coalescence of emulsions or Ostwald ripening, the area of the entire interface decreases, resulting in a decrease in the storage modulus (G′) [22]. This phenomenon was also observed in the CNC-based PEs. The stability of the PE was evaluated by increasing the CNC concentration from 45 mg/mL to 180 mg/mL by performing frequency sweeps. Emulsions with less than 90 mg CNC/mL EO showed a large particle size and wide distribution of particle sizes. As the size of the PE increased, the interactions between PEs decreased, leading to a decrease in G′. Increased PE size also reduced the regularity of the emulsion arrangement in the system, inducing the lower G′ value. The PEs prepared with more than 135 mg CNC/mL EO had a smaller emulsion size and narrower distribution than the other PEs, implying a more regular arrangement of emulsion particles. In particular, the PEs with 180 mg CNC/mL EO showed a constant strain value of 0.5% and a G′ value of about 5.5 Pa over the entire frequency range.

EOs are available from the natural resources and become continuously popular in biological and medical applications due to their antimicrobial effect. Despite the significant biological activity of EOs, water insolubility limits their use as a generic antibiotic agent. In recent years, oil in water-type PEs stabilized with nanoparticles including silica, graphene and CNC have been prepared, and the water solubility of oils has been improved. Thyme white EO is known in the research to cause the microbial cytoplasm to crack, and the thymol and carvacrol in it destroy microbial metabolic organelles or cause leakage of internal organs [4,48,49].

PE for the investigation of biological activity was prepared with 135 mg CNC/mL EO by tip sonication considering the uniformity of the emulsion droplets and narrow size distribution. The antimicrobial activity of thyme white only and PEs containing thyme white EO was determined by MIC and MBC against *E. coli* and *S. aureus* using streptomycin as a positive control (Table 2, Table 3, Table 4 and Table 5).

Thyme white only showed weak antimicrobial activity against *E. coli* and no microbial growth or turbidity was observed over 0.625 μL/mL, indicating the MIC value. On the other hand, CNC/EO PEs showed strong activity against *E. coli* at 0.312 μL/mL, which was a lower MIC value than thyme white only, demonstrating the efficiency of antimicrobial activity. For *S. aureus*, the microbial growth was also inhibited by thyme while only at MIC 0.625 μL/mL and CNC/EO PEs at MIC 0.250 μL/mL (Table 2 and Table 3).

The lowest concentration of an antimicrobial agent for microbial death (MBC) was demonstrated as a complementary to the MIC. For *E. coli*, MBC was 1.00 with thyme white only and 0.750 with CNC/EO PEs. For *S. aureus*, MBC was 1.00 with thyme white only and 0.650 with CNC/EO PEs (Table 4 and Table 5). Streptomycin showed no microbial growth at the concentrations used in the investigation.

**Figure 6:**
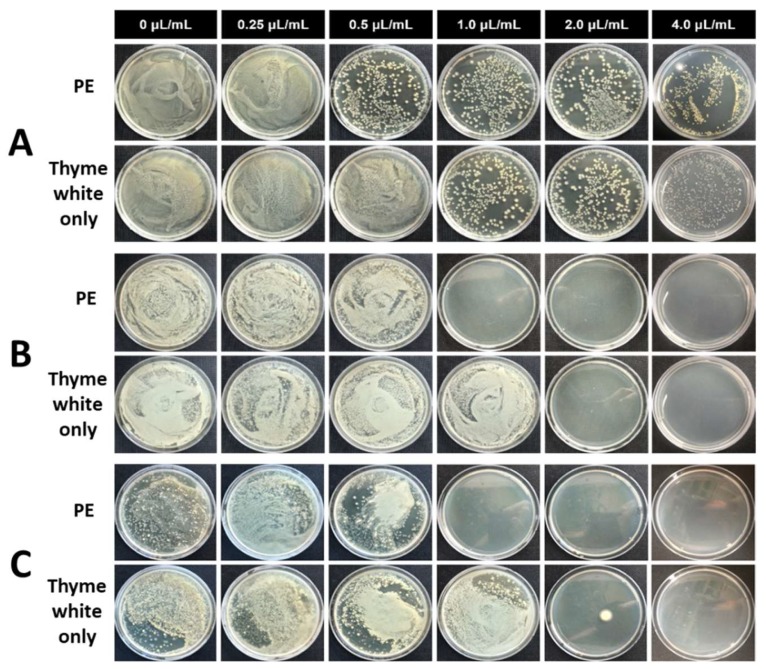
Antimicrobial activity of CNC/EO PEs and thyme white only against *E. coli* at different concentrations of EO. The extracts were diluted 1000 times and spread on an agar plate after the storage for (**A**) 0 d, (**B**) 10 d, and (**C**) 25 d.

**Figure 7:**
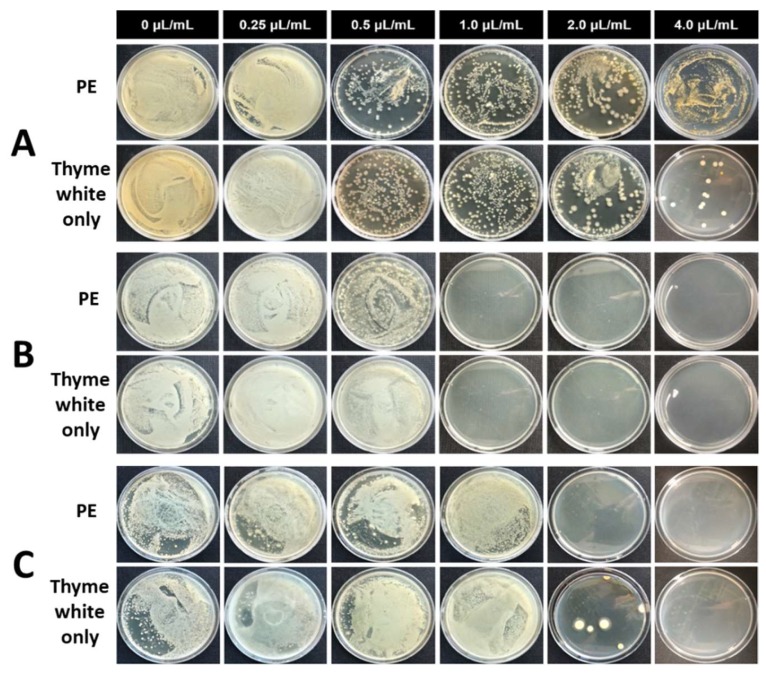
Antimicrobial activity of CNC/EO PEs and thyme white only against *S. aureus* at different concentrations of EO. The extracts were diluted 1000 times and spread on an agar plate after storage for (**A**) 0 d, (**B**) 10 d, and (**C**) 25 d.

For the investigation of long-term antimicrobial activity, the different contents of PE and thyme white only were added to the medium containing microbes, and the mixture was incubated up to 25 d. The incubated solution was sampled, diluted and spread over the surface of the solid agarose gel medium. The antibacterial activity of PE and thyme white only was evaluated by counting the number of colonies at the gel surface after incubation for 3 d (Figure 6 and Figure 7). PE showed antimicrobial activity with 1.0 μL/mL EO against *E. coli* (Figure 6A–C). The microbes were bioactive immediately after the addition of PE, but no colony was observed with the extractions incubated for 10 d and 25 d. However, thyme white only showed microbial activity with the extractions incubated for 10 d and 25 d at the same concentration. The bioactivity of *S. aureus* was also restricted by the addition of PE with 1.0 μL/mL EO or more (Figure 7A–C). The dense CNC shell reduced the volatility of EO and prevented the rapid release of EO into the solution. As a result, the CNC enabled longer and sustained antimicrobial activity in the bulk environment.

The larvicidal activity of the thyme white oil and two types of formulation is shown in Table 6. Thyme oil and CNC-based formulation of thyme white oil showed 100% larvicidal activity against *Ae. albopictus* at a concentration of 0.1 mg/mL. CNC-based formulation of thyme white oil exhibited 100% larvicidal activity at 0.05 mg/mL concentration, but no mortality was observed in thyme oil. However, the larvicidal activity of the CNC-based formulation of thyme white oil was weaker than that of temephos. This result indicated that the thyme white essential oil had been successfully liberated from the thyme white essential oil-loaded formulations. The larvicidal activity of thyme white oil-loaded formulations was stronger than that of thyme white oil. This might be attributed to high solubility in water of thyme white oil-loaded formulations. Plant essential oil is highly hydrophobic, and this is one obstacle for the development of plant essential oil-based mosquito larvicides. Our results showed that the CNC-based formulation could solve the insolubility problem of plant essential oil in water. Another reason for the high activity of the CNC-based formulation might be attributed to the controlled release of thyme white oil. Plant essential oil easily evaporated when applied in water [50]. High mortality of CNC-based thyme white oil formulation might be achieved by the controlled release of thyme white oil over the desired period. Development of proper formulation for practical use of plant essential oil in field has been conducted [19,50]. Osanloo et al. [19] found that nanoemulsions of plant essential oils enhanced the larvicidal activity, stability and solubility of plant essential oils. Seo et al. [50] also reported that parsley oil-loaded PVA microemulsion showed strong larvicidal activity against *Ae. albopictus* and could solve the insolubility problem of plant essential oils in water.

## 4. Conclusions

CNCs with a needle-like morphology were produced by sulfonic acid hydrolysis. These CNCs showed surface active ability for the formation of PE with thyme white EO. A critical concentration of the CNC-based PEs was determined to be 135 mg CNC/mL EO. Smaller emulsion particles resulted in a higher G′ value, implying a more regular arrangement of emulsion particles and dimensional stability. The size of CNC/EO PE varied according to the CNC content in the emulsifying process. The size of PE particles decreased as the CNC content increased. Encapsulation of EO in CNC shell was visualized by distinct fluorescent labelling. The size of PE was about 5 μm for concentrations over 135 mg CNC/mL EO with comparatively narrower size distributions. The antimicrobial ability of CNC/EO PE was confirmed by the determination of minimum bactericidal concentration with long-term storage up to 25 d. The larvicidal activity of CNC/EO PE was stronger than that of pure EO due to high solubility and sustainability of EO in water.

## Figures and Tables

**Table 1:** Chemical composition of thyme white essential oil

Compounds	Retention Indices		Composition Rate (%)
	DB-1MS	HP-Innowax	
*α*-Pinene	928	1021	7.22
Camphene	940	1064	1.32
*β*-Pinene	967	1108	0.66
*β*-Myrcene	982	1165	0.96
α-Phellandrene	994	1165	0.16
*p*-Cymene	1010	1275	38.14
Limonene	1020	1200	7.93
*γ*-Terpinene	1050	1248	0.38
Linalool	1088	1538	3.57
*α*-Terpineol	1170	1707	0.25
Thymol	1273	2207	35.82
Carvacrol	1278	2235	0.45
Sum	-	-	93.29

**Table 2:** Minimal inhibitory concentration (MIC) of PE, thyme white only solution and streptomycin against *E. coli*. ^1^

Dilution of Antimicrobial Compounds for Same No. of Bacteria (μL/mL)	Antimicrobial Activity (MIC, *Escherichia coli*)							
	0.125 ^2^	0.150	0.250	0.312	0.500	0.625	1.000	1.250
Thyme White Only	+	+	+	+	+	-	-	-
CNC/EO PEs	+	+	+	-	-	-	-	-
Streptomycin	-	-	-	-	-	-	-	-

Positive (+) = Turbidity indicating growth; Negative (-) = No turbidity indicating absence of growth ^1^ Results are means of three replicates, ^2^ μL/mL

**Table 3:** Minimal inhibitory concentration (MIC) of PE, thyme white only solution and streptomycin against *S. aureus*. ^1^

Dilution of Antimicrobial Compounds for Same No. of Bacteria (μL/mL)	Antimicrobial Activity (MIC, *Staphylococcus aureus*)							
	0.125 ^2^	0.150	0.250	0.312	0.500	0.625	1.000	1.250
Thyme White Only	+	+	+	+	+	-	-	-
CNC/EO PEs	+	+	-	-	-	-	-	-
Streptomycin	-	-	-	-	-	-	-	-

Positive (+) = Turbidity indicating growth; Negative (-) = No turbidity indicating absence of growth ^1^ Results are means of three replicates, ^2^ μL/mL.

**Table 4:** Minimal bactericidal concentration (MBC) of PE, thyme white only solution and streptomycin against the *E. coli*. ^1^

Dilution of Antimicrobial Compounds for Same No. of Bacteria (μL/mL)	Antimicrobial Activity (MBC, *Escherichia coli*)								
	0.600 ^2^	0.650	0.700	0.750	0.800	0.850	0.900	0.950	1.000
Thyme White Only	+	+	+	+	+	+	+	+	-
CNC/EO PEs	+	+	+	-	-	-	-	-	-
Streptomycin	-	-	-	-	-	-	-	-	-

Positive (+) = Indicating growth; Negative (-) = Indicating absence of growth ^1^ Results are means of three replicates, ^2^ μL/mL.

**Table 5:** Minimal bactericidal concentration (MBC) of PE, thyme white only solution and streptomycin against the *S. aureus*. ^1^

Dilution of Antimicrobial Compounds for Same No. of Bacteria (μL/mL)	Antimicrobial Activity (MBC, *Staphylococcus aureus*)								
	0.600 ^2^	0.650	0.700	0.750	0.800	0.850	0.900	0.950	1.000
Thyme White Only	+	+	+	+	+	+	+	+	-
CNC/EO PEs	+	-	-	-	-	-	-	-	-
Streptomycin	-	-	-	-	-	-	-	-	-

Positive (+) = Indicating growth; Negative (-) = Indicating absence of growth ^1^ Results are means of three replicates, ^2^ μL/mL.

**Table 6:** Larvicidal activities of thyme white EO and CNC/EO PEs against *Ae. albopictus*.

Compounds	Larvicidal Activity (%, Mean ± S.E, N = 4)		
	0.1 ^1^	0.05	0.025
Thyme White Only	100	0	- ^2^
CNC/EO PEs	100	100	38.0 ± 8.0
Ethanol Only	0	0	0
CNC Only	0	0	0
Temephos	100	100	100

^1^ mg/mL, ^2^ Not tested.
